# Bamboo ecosystem services in 25 years: a systematic literature review of trends, insights, and knowledge gaps

**DOI:** 10.1007/s11356-025-36650-7

**Published:** 2025-06-25

**Authors:** Chong-En Li, Shih-Yu Lee, Yi-Ying Chen, Shih-Yun Kuo, Mei-Hua Yuan

**Affiliations:** 1 Research Center for Environmental Changes, Academia Sinica, Taipei City, Taiwan; 2 National Science and Technology Center for Disaster Reduction, New Taipei City, Taiwan

**Keywords:** Synergy, Trade-off, PRISMA, Forest management

## Abstract

While bamboo offers various benefits to people, existing reviews on the current state of knowledge and research trends regarding its ecosystem services remain limited. This study aims to systematically review the literature on bamboo ecosystem services, focusing on temporal and geographic patterns, thematic trends, and methodological approaches, as well as the exploration of synergies and trade-offs. It adheres to the Preferred Reporting Items for Systematic Reviews and Meta-Analyses 2020 guidelines and includes an analysis of 56 relevant studies indexed in Scopus. This review shows a recent growing academic interest in bamboo ecosystem services. Research is primarily concentrated in regions where bamboo is commonly found, shaped by regional research capacity, and characterized by both locally led efforts and externally conducted studies. Keyword and co-occurrence analyses identify three main research clusters focused on the quantitative assessment, qualitative investigation and economic valuation of ecosystem services. Methodologically, studies tend to rely on plot sampling for data collection, apply indices for analysis, and present findings through mapping and statistical techniques. Among ecosystem service categories, regulation and maintenance services receive the most attention. However, interactions among services remain underexplored, with research on synergies and trade-offs still limited. These findings provide valuable insights for bamboo forest management and indicate future research directions that merit deeper exploration.

## Introduction

Human dependence on ecosystems is fundamental to our existence. The concept of ecosystem service (ES) has become a prominent topic in contemporary academic discussions. This term refers to the benefits that humans derive from ecosystem functions. Since the Millennium Ecosystem Assessment introduced the first widely accepted framework (Reid et al. 2005), an increasing number of classification systems have been proposed. Nevertheless, their core structures remain largely consistent, such as frameworks proposed by Beaumont et al. (2007), the Economics of Ecosystems and Biodiversity (TEEB; Kumar 2010), and the Common International Classification of Ecosystem Services (CICES; Haines-Young and Potschin 2018). These frameworks serve as valuable tools for comprehensively understanding the multifaceted contributions of ecosystems to human well-being.

Bamboo is a highly versatile woody grass recognized for its rapid growth and strong renewability (Chaudhary et al. 2024). These characteristics contribute to its remarkable capacity for carbon sequestration (Kuehl et al. 2013; Li et al. 2015). Beyond this, bamboo also provides diverse ESs shaped by its unique ecological traits. However, research on the ESs of bamboo remains underexplored compared to other species (Paudyal et al. 2019). This knowledge gap is particularly concerning given the geographic distribution of bamboo, predominantly in tropical and subtropical regions where socioeconomic development is often limited. In such contexts, natural resources are frequently subject to overexploitation, habitat degradation, and poor management (Geist and Lambin 2002). Consequently, bamboo might be cut down by local communities in large numbers before their value is fully realized (Embaye 2001). This highlights an urgent need to identify bamboo’s contributions to improving forest management.

Given the limited number of existing reviews on the ESs provided by bamboo, we argue that a thorough exploration of related trends, insights, and knowledge gaps is warranted. Specifically, we investigate the temporal and spatial distribution of studies, analyze contributor and funding characteristics, examine the keywords and methodologies employed, and identify the ESs discussed in the literature while reviewing their interactions. These research objectives are designed to inform bamboo forest management and future research directions. The following sections are organized accordingly: the “Materials and methods” section details research materials and methods. The “Results” section presents the results of the systematic literature review, which are discussed in the “Discussion” section. Finally, the “Conclusion” section synthesizes the findings and provides conclusions.

## Materials and methods

The systematic literature review offered a comprehensive and integrative overview of the current state of knowledge and research trends. In conducting this review, we followed the Preferred Reporting Items for Systematic Reviews and Meta-Analyses (PRISMA) 2020 Guidelines, which provide a transparent and replicable process for identifying, screening, and including relevant studies while minimizing potential bias. PRISMA has been adopted in numerous scientific literature reviews, including those exploring ES topics (e.g., Liquete et al. 2013; Ladino et al. 2019; Perevochtchikova et al. 2019; Carucci et al. 2022; Chalkiadakis et al. 2022; Van der Hoff et al. 2022). Two independent reviewers were involved in identifying potentially relevant studies and then jointly screened the articles to determine inclusion. The review was limited to English, Mandarin, and Japanese publications, available before May 19, 2023. Gray literature, such as conference papers, reviews, editorials, and letters, was excluded to maintain academic rigor.

**Fig. 1:**
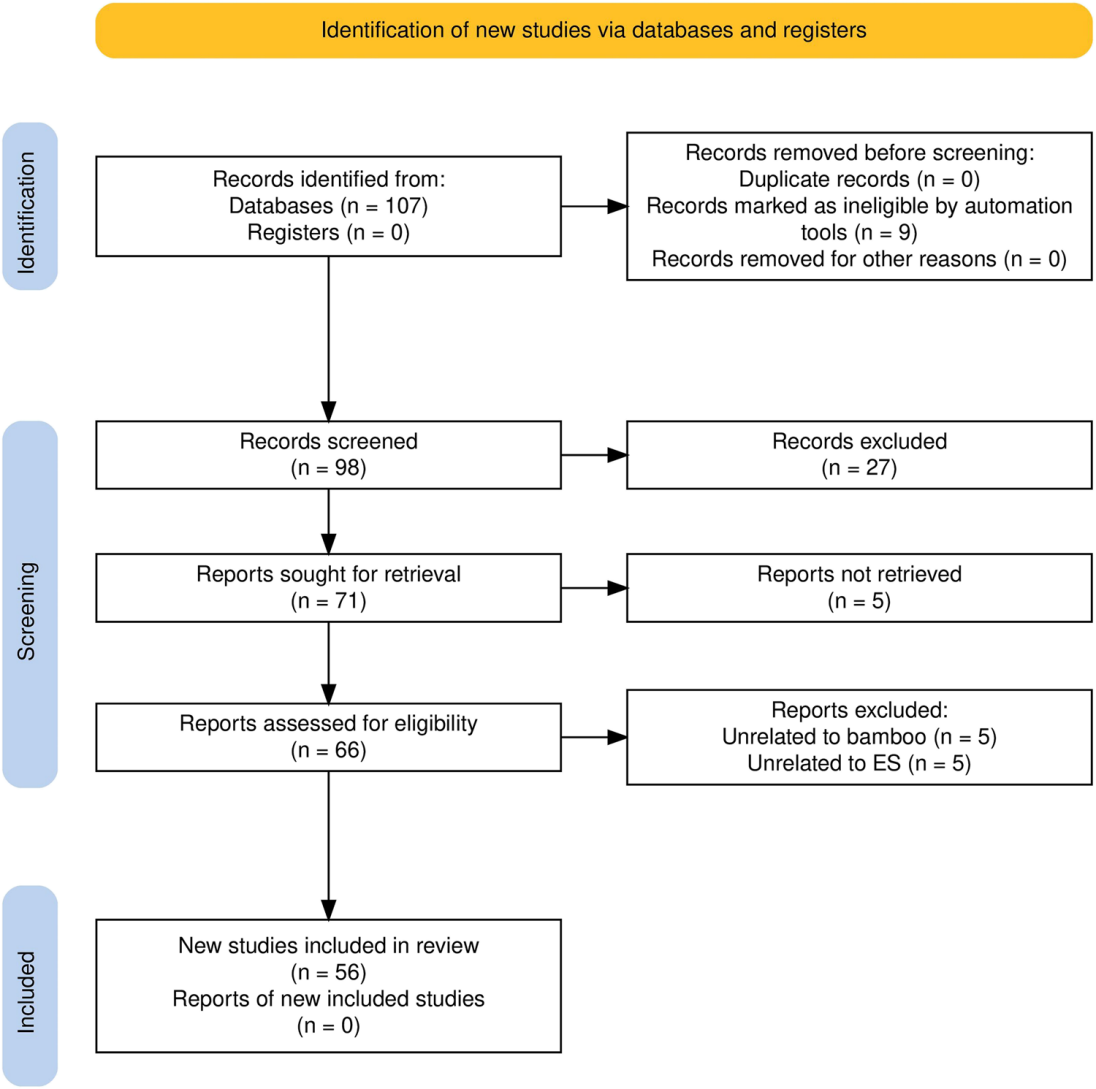
PRISMA 2020 flow diagram generated using https://estech.shinyapps.io/prisma_flowdiagram/ (Haddaway et al. 2022)

The literature search was conducted using the Scopus website (https://www.scopus.com/), selected due to its status as the “world’s largest abstract and indexing database” of peer-reviewed literature covering scientific, technical, medical, and social science disciplines. We used the keywords “bamboo” and “ecosystem service” to search within the article title, abstract, and keywords fields. Figure 1 illustrates the procedure for selecting articles through a PRISMA 2020 flow diagram. Initially, we identified 107 results from Scopus, and nine records were removed based on their document type and language filters. Subsequently, we read the abstracts of the 98 remaining studies and excluded 27 studies that independent reviewers determined to be unrelated to bamboo ES during the screening stage. We further excluded five articles since their full texts were not available. Upon a deeper review of the full texts of the articles, ten records were excluded based on our predefined criteria. In the final stage, we compiled the fundamental information of the 56 remaining studies into a table, which served as the original database for this review. The information extracted included the article titles, year of publication, authors’ names and affiliations, funding sources, affiliation countries of authors, analytical scales, study areas, keywords, journal titles, research techniques and tools, and the identified ESs and the mentioned synergies and trade-offs. The selected publications form the basis for the subsequent bibliometric and thematic analyses in the following sections.

## Results

### Publication trends

**Fig. 2:**
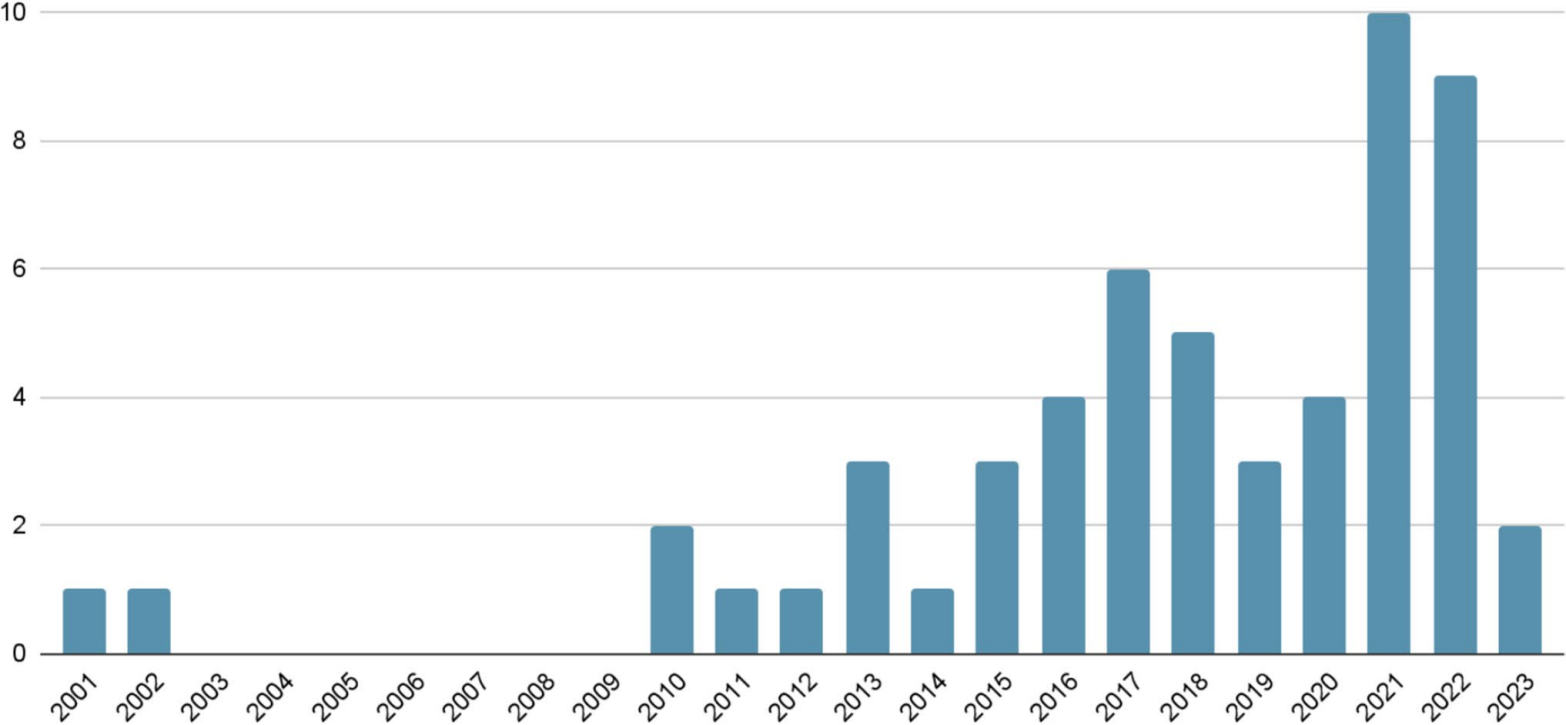
Year of the selected publications

A total of 52 journal articles and four book chapters were included in this review. Analysis of the publication years reveals notable developmental trends. Initially, research on bamboo-related ESs began with two publications in 2001 and 2002, conducted by Swedish and Indian researchers in Ethiopia and India, respectively. However, no publications were recorded over the subsequent 7 years. Interest in this field reemerged in the early 2010s, with an average of approximately three publications per year throughout that decade. Research output has grown notably in recent years, reaching a peak of ten publications in 2021. This upward trend highlights the emerging and evolving nature of bamboo ES research and suggests a growing academic interest in the topic (Fig. 2).

Despite the growing number of publications on ESs, the field of bamboo ES research remains highly fragmented in terms of authorship, institutional affiliations, and funding sources. No individual researchers have maintained a sustained presence across multiple studies, and there is a notable absence of dominant research centers or leading agencies. This lack of continuity among contributors suggests that scholarly engagement in the field is still scattered and largely exploratory. In other words, a cohesive academic community that consistently produces Scopus-indexed research in this domain has yet to emerge.

### Geographic patterns

**Fig. 3:**
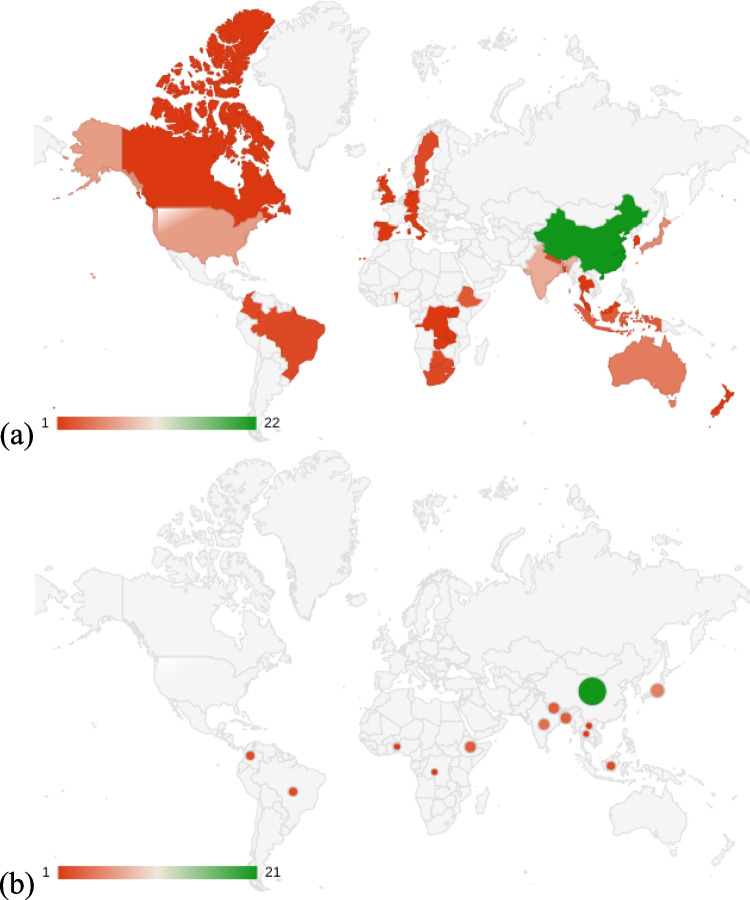
Countries of **a** authors’ affiliations and **b** study areas among selected publications

Approximately 80% of the world’s bamboo forests are in Asia, with the highest concentrations in China and India (Lobovikov et al. 2012). Given this background, we analyzed the geographical patterns of bamboo ES research by examining both the institutional affiliations of authors and the geographic locations of study areas (Fig. 3). The results indicate that author affiliations were predominantly from China (*n* = 22), followed by Japan (*n* = 5) and India (*n* = 4). In terms of study areas, most studies (*n* = 40) were conducted at a local analytical scale, while five studies each were conducted at subnational and national scales. Six publications did not specify a particular spatial scale. Study areas were largely concentrated in China, India, and several Southeast Asian and African countries which closely mirror the global distribution of bamboo forests.

To further investigate the relationship between author affiliations and study locations, we classified the selected publications into three categories: (1) studies conducted by Chinese researchers within China (*n* = 21); (2) studies carried out by Western or local researchers focusing on developing countries (*n* = 23); and (3) studies conducted by researchers from developed countries within their own national contexts (*n* = 5), primarily in Japan.

### Keyword analysis

**Fig. 4:**
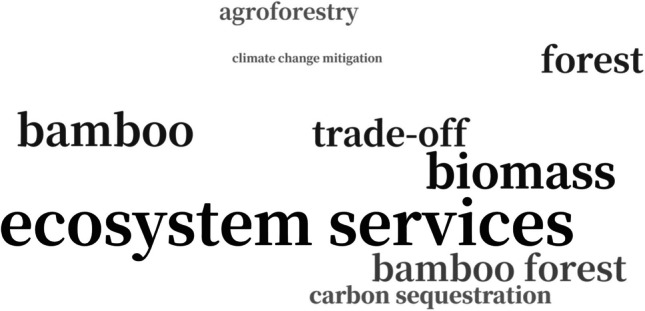
Word cloud of keywords from the selected publications

**Fig. 5:**
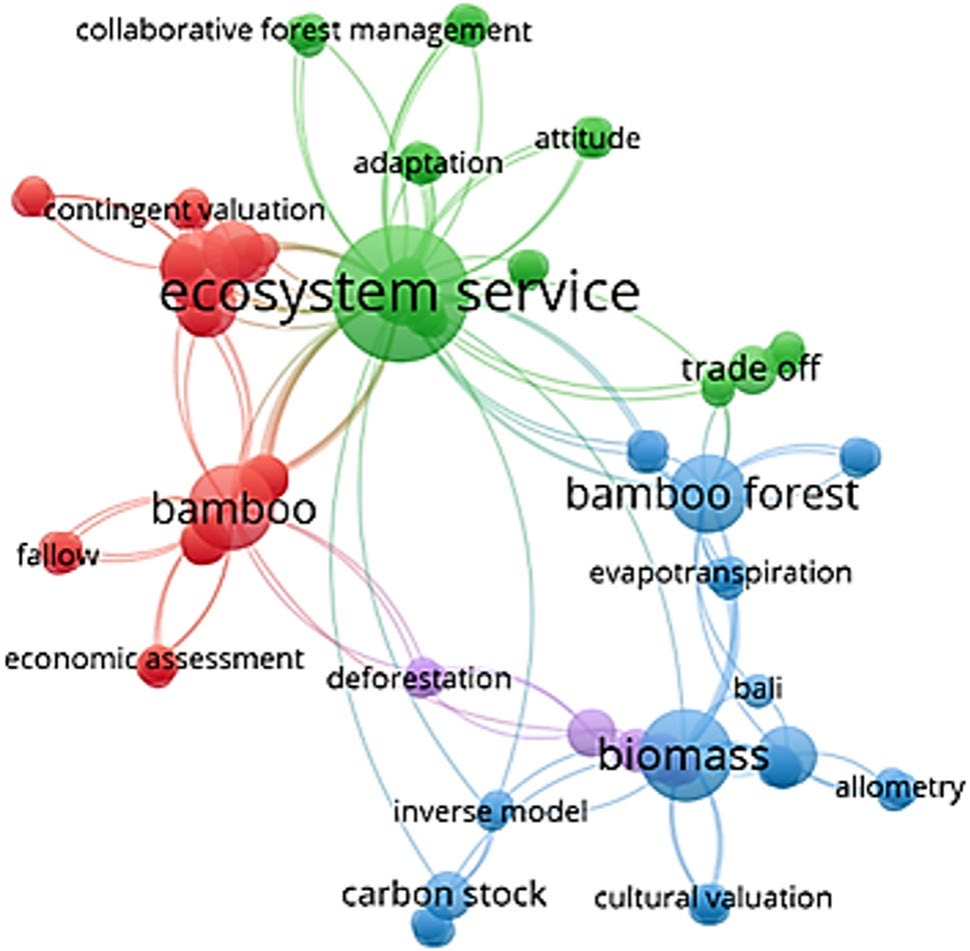
Co-occurrence network of keywords from the selected publications

Keyword analysis can provide valuable insights into current research interests. Figure 4 presents a word cloud generated from the keywords of the selected publications, displaying only those terms that appeared more than three times. Keywords from three articles were unavailable and the remaining keywords were cleaned and standardized using a synonym dictionary. As anticipated, “ecosystem service” and “bamboo” emerged as the most prominent keywords, consistent with the initial search criteria. Other frequently observed terms include “biomass,” “trade-off,” and “carbon sequestration,” reflecting researchers’ interest in bamboo ESs and their interactions. To further investigate the relationships among these keywords, a co-occurrence network analysis was performed using VOSviewer (version 1.6.19; Leiden University). This approach provides a visual representation of keyword frequencies and their linkages, allowing the identification of dominant research themes and detecting emerging trends across the field (Dotsika and Watkins 2017; Lozano et al. 2019). The resulting network diagram reveals three primary thematic clusters (Fig. 5). Blue cluster centers on quantifying the ESs of bamboo, with “biomass” and “carbon stock” as representative keywords. Green cluster focuses on qualitative research dimensions, incorporating terms such as “attitude” and “adaptation.” Red cluster highlights economic evaluation methods, including “economic assessment” and “contingent valuation.”

We observed that the blue cluster predominantly comprises studies conducted by Chinese researchers, who often adopt quantitative, data-driven methodologies. Representative works include Wang et al. (2011), Pan et al. (2012), Huang et al. (2018), Cui et al. (2019), published in the journal “*Acta Ecologica Sinica*,” as well as Zhuang et al. (2022), Feng et al. (2022), Ge et al. (2022), published in the journal “*Forests*.” Additional examples include Hu et al. (2013), Liu et al. (2013), Li et al. (2015), Dai et al. (2017), Wu et al. (2020), Li et al. (2020), Dai et al. (2022), Ma et al. (2022), and Xiao et al. (2023). Notably, in the selected publications, all authors who submitted to the above-mentioned two journals were affiliated with institutions in China. These studies consistently adopt quantitative methodological approaches, reflecting the prevailing research paradigm in China. *Acta Ecologica Sinica*, published by Elsevier, is a journal in China exclusively devoted to ecology and its sub-disciplines. *Forests* is an international journal focusing on forestry and forest ecology, published by MDPI.

In contrast, research from other developing countries tends to rely on qualitative approaches, as seen in the green cluster, focusing on local perceptions, cultural contexts, and participatory engagement. This methodological choice is often a practical response to limited data availability, especially when researchers are not native to the study site. As Paudyal et al. (2015) suggest, techniques such as semi-structured interviews, focus group discussions, transect walks, and participatory mapping are particularly suited to identifying and assessing ESs in data-scarce contexts. Representative works in this category include Saxena et al. (2002), Okubo et al. (2010), Gao et al. (2013), Ingale and Deore (2015), Houdanon et al. (2018), Ahammad et al. (2019), Baul et al. (2021), Gurung et al. (2021), and Ndavaro (2023).

### Research techniques and tools

**Fig. 6:**
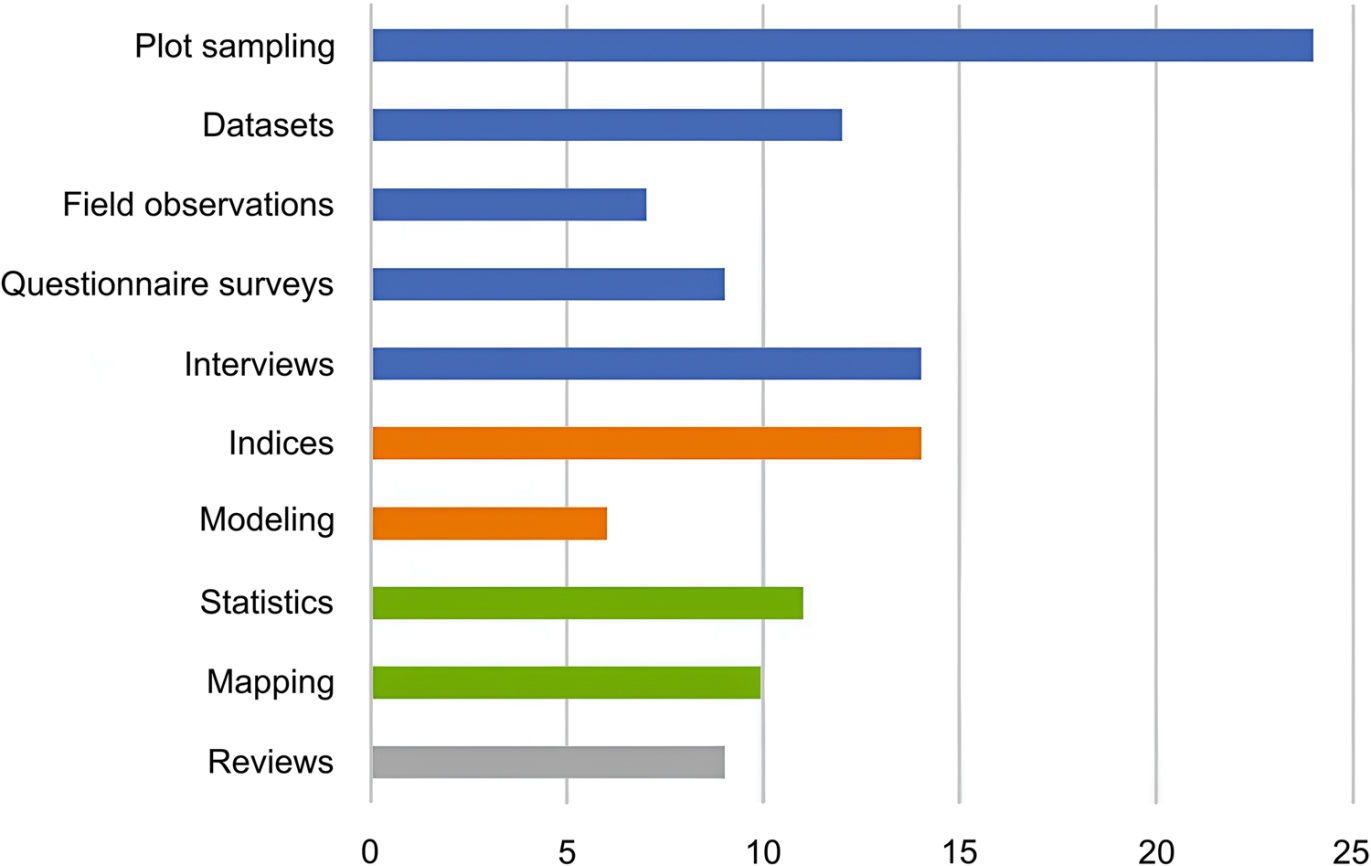
Number of selected publications employing each analytical technique

Reviewing existing research techniques and tools can provide valuable insights for designing future study. In our review of selected publications, we identified ten distinct techniques: plot sampling, datasets, field observations, questionnaire surveys, interviews, indices, modeling, statistics, mapping, and reviews. The definitions of each technique are presented in Table 1, while their frequency of use across studies is illustrated in Fig. 6. As individual studies often employ multiple techniques, the total number exceeds the number of reviewed publications.

**Table 1:** Definitions of analytical techniques used in the selected publications

Techniques		Meaning
Data collection	Plot sampling	Established areas for collecting quantitative data
	Datasets	Quantitative secondary data, aerial or satellite images from existing databases, which are often released by the governments
	Field observations	Qualitative data collection through direct viewing in the research area
	Questionnaire surveys	(Semi-)structured means to gain qualitative data
	Interviews	Unstructured means to gain qualitative data
Data analysis	Indices	Functions or formulas served to calculate specific parameters, such as Shannon Wiener Index
	Modeling	Estimate ES by widely recognized frameworks (e.g., InVEST model)
Illustration	Statistics	Representing results by descriptive or inferential statistics
	Mapping	Representing results by map
Reviews		Encompasses a critical review or examination of previous literature

Sources: Refer to Logsdon and Chaubey (2013), Rasmussen et al. (2016), and Chalkiadakis et al. (2022)

According to the results, data collected using quantitative methods were more prevalent than data obtained through qualitative approaches. Among the quantitative studies, plot sampling was more frequently employed than the use of secondary datasets. Notably, all studies utilizing existing datasets were conducted by Chinese researchers, suggesting that their greater accessibility to open datasets may play a decisive role. However, some small-scale Chinese studies also employed plot sampling (Pan et al. 2012; Liu et al. 2013; Yu et al. 2016; Li et al. 2017; Wu et al. 2020; Li et al. 2020; Feng et al. 2022; Ge et al. 2022; Xiao et al. 2023). Combined with the fact that researchers from other countries, facing data scarcity, also had no choice but to adopt plot sampling in their quantitative studies, this contributed to establishing it as the most commonly used method overall.

In terms of data analysis techniques, indices were more frequently employed than modeling approaches. Specifically, 16 quantitative studies utilized indices (e.g., Hu et al. 2013), whereas only six studies adopted modeling tools for data estimation and analysis. These tools included the InVEST model (Dai et al. 2017, 2022; Feng et al. 2022; Ma et al. 2022), the CASA model (Dai et al. 2017, 2022), the Modified-Hargreaves model (Feng et al. 2022), the BEPS model (Li et al. 2020), and a modified model based on the SCS-CN method (Lin et al. 2017). Notably, all studies applying the technique of modeling were conducted by Chinese researchers and were published in recent years. This trend may once again reflect the greater accessibility to open datasets in China, which in turn facilitates the adoption of advanced modeling techniques. In contrast, researchers from other countries may encounter data constraints that limit their ability to implement this technique. Furthermore, regardless of the quantitative technique employed, visual representation of results through mapping remains a highly effective communication strategy. However, only five of the quantitative articles utilized mapping to present their findings (Wang et al. 2011; Li et al. 2015, 2020; Dai et al. 2017; Cui et al. 2019).

As for qualitative research, interviews were the most popular technique, followed by questionnaire surveys and field observations. These qualitative research methods were primarily used in developing countries (excluding China), in contrast to China, where studies mainly conducted quantitative research. It is worth noting that many of these studies were not purely qualitative, and most of the selected publications adopted a mixed methods approach. Those articles collected qualitative data through interviews or questionnaire surveys and then analyzed the data using statistical methods. This pattern reflects a common trend in social research, where qualitative insights are increasingly integrated with statistical tools (Denscombe 2008). These mixed method studies used stratified sampling based on factors such as village location (Gao et al. 2013), wealth (Ahammad et al. 2019), community/expert (Lee 2021), homestead forest size (Yeasmin et al. 2021), or combined criteria (Baul et al. 2021) to compare the amount of ESs obtained across different groups. Some studies also apply regression analysis to further explore these relationships (Okubo et al. 2010; Gao et al. 2013).

In addition to the aforementioned techniques, literature reviews have become increasingly common, with 12 selected publications employing this technique. Review articles often yield broader insights into bamboo ESs compared to case studies, as demonstrated in the works of Embaye (2001), Shinohara et al. (2014), and Brown et al. (2018).

### Identified service categories

**Fig. 7:**
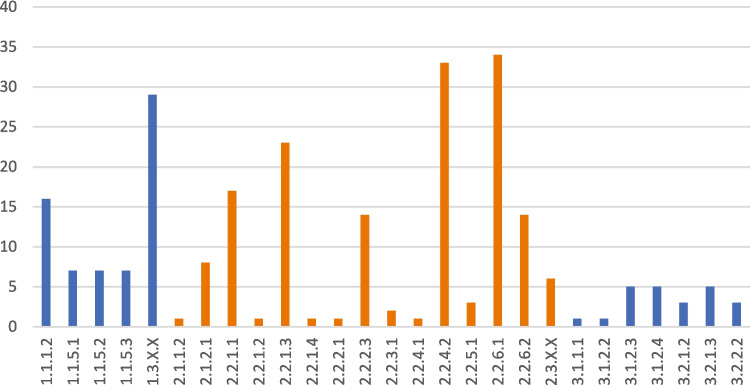
Types of ESs identified from the selected publications

The CICES provides a concrete standard to categorize ES. In its version 5.1 framework, Haines-Young and Potschin (2018) organized ESs into three main categories. The provisioning service “covers all nutritional, non-nutritional material, and energetic outputs from living systems as well as abiotic outputs (including water).” The regulation and maintenance services are “all the ways in which living organisms can mediate or moderate the ambient environment that affects human health, safety, or comfort, together with abiotic equivalents.” Finally, the cultural services are “all the non-material, and normally non-rival and non-consumptive, outputs of ecosystems (biotic and abiotic) that affect physical and mental states of people.” Within each category, ESs are classified more precisely through a hierarchical structure composed of four levels: division, group, class, and class type. These levels together generate a unique CICES code for each ES (Appendix Table 2). Given its widespread adoption, this framework became one of the standard systems for identifying and classifying ESs. Our independent reviewers evaluated selected publications and applied the classification of ES following the CICES V5.1 guidelines. The analysis revealed that regulation and maintenance services were the most frequently addressed in the selected publications (*n* = 97), followed by provisioning services (*n* = 17). In contrast, cultural services were the least discussed (*n* = 11). Figure 7 presents the classification of each ES mentioned in the selected articles according to its corresponding CICES code. Among them, ES types related to animals (CICES codes was 1.1.3.1–⁠1.1.4.3, 1.1.6.1–⁠1.1.6.3, and 1.2.2.1–⁠1.2.2.3) and abiotic (CICES codes was 4.2.1.1–⁠6.3.X.X) were omitted. Definitions of the CICES codes are provided in Appendix Table 2﻿, while Appendix Table 3 provides a detailed list of the selected articles associated with each CICES code. Notably, although bamboo forests possess unique ecological characteristics, many studies assess ESs only in the context of general forest ecosystems without specifically distinguishing bamboo. As a result, several studies listed in Appendix Table 3 focus on general forests rather than bamboo-specific ecosystems.

### Synergies and trade-offs

Synergies are defined “as the positive response of multiple ESs to a change in the driver” (Bennett et al. 2009), while a trade-off is described as “an antagonistic situation that involves losing one quality of something in return for gaining another” (Cord et al. 2017). To understand the relationships between synergies and trade-offs in bamboo ESs, we reviewed the selected publications for relevant information. However, consistent with the observation by Bennett et al. (2009), most of the reviewed studies assessed ESs in isolation and did not explicitly analyze their interactions. To better interpret the few that did, we classified the articles into three levels based on the depth of discussion. At the most basic level, five studies merely examined the advantages and disadvantages of bamboo relative to other species, often focusing on a single ES. For instance, Pan et al. (2012) showed that shrub and grassland soils have stronger soil water-holding capacity than general and bamboo forests. Huang et al. (2018) argued that the ES of bamboo is the least among other tree species. In Japan, Moso bamboo invades neighboring forests by stretching subterranean stems that seriously damage the landscape (Kamada 2017) and prevent sunlight from reaching the forest floor (Hasegawa et al. 2021). Visitors at an alpine site appreciate snow-covered terrain and distinctive flora, but participant responses indicate that dwarf bamboo encroachment has significantly reduced the landscape’s aesthetic appeal (Mameno et al. 2022). Strictly speaking, however, such comparisons do not constitute analyses of synergies or trade-offs, which require an explicit assessment of interactions among multiple services.

At the second level, six studies explicitly identified synergies and trade-offs between two specific ESs. For instance, Embaye (2001) argued that bamboo is being heavily logged in Ethiopia to expand agricultural land, leading to rapid ecological deterioration, with land productivity declining yearly. Okubo et al. (2010) mentioned that maintaining biodiversity in bamboo plantations may not provide sufficient income for bamboo plantation owners. Panthi et al. (2017) found that increased bamboo harvesting may lead to further habitat degradation through loss of forage and associated human disturbance. The above studies identified bamboo harvesting as the driver that leads to trade-offs among specific ES. Conversely, some studies considered bamboo removal as the driver but discovered synergy relationships. For instance, Katayama (2020) found that annual harvesting is necessary to increase the productivity of edible bamboo culm. Ge et al. (2022) argued that Moso bamboo tends to cause unconstrained expansion into adjacent woodlands and adverse effects on the function services of forest ecosystems, leading to high biomass mortality and lower standing biomass, reducing forests’ carbon stocks over time (Altomare et al. 2022), and decline in the diversity of nematodes (Xiao et al. 2023). Besides agriculture, animal husbandry is also a pivotal factor in affecting bamboo forest ecosystems. Li et al. (2017) pointed out that raising horses reduces bamboo and affects the panda habitat.

Nevertheless, most species provide more than two types of ESs, which are often interlinked, as each service can influence or reinforce others in complex ways. Studies examining interactions among more than three ESs were categorized as belonging to the third analytical level. Researchers have employed various methods to capture these complex interrelations, including descriptive approaches and correlation analysis. An example of the former is Houdanon et al. (2018), who described synergistic relationships among bamboo used for craft material, firewood, and cultural dance. The example of the latter include Egoh et al. (2008) and Pan et al. (2012), who calculated correlation coefficients between different ESs and visualized them using matrix tables.

Recent research has emphasized the importance of spatial distribution in identifying synergies and trade-offs (Prudencio and Null 2018), highlighting that spatial heterogeneity plays a crucial role in shaping ES interactions. For instance, Chang et al. (2021) showcased how mapping can enhance urban green infrastructure planning by visualizing the spatial dynamics of ES interactions, which matrix tables alone cannot adequately capture. However, this method was explicitly applied to bamboo-related ESs in only three of the studies reviewed (Dai et al. 2022; Feng et al. 2022; Ma et al. 2022). In addition to spatial considerations, temporal dynamics have also been emphasized. Tomscha and Gergel (2016) noted that ES interactions are often inferred solely from spatial patterns, thereby overlooking important temporal dimensions. Considering temporal variation enables researchers to better understand evolving synergies and to anticipate or mitigate trade-offs. However, only a few selected publications assessed historical changes (Wu et al. 2020; Li et al. 2020) and simulated potential future scenarios (Li et al. 2015; Ma et al. 2022).

## Discussion

### Implications for management

The first finding of this study reveals a growing academic interest in the ESs offered by bamboo ecosystems. This trend underscores the opportunity for policymakers and land managers to integrate emerging scientific insights into current bamboo management strategies. Such integration can improve the sustainable governance of bamboo forests by applying advanced approaches. To this end, we suggest several actionable strategies, particularly those that leverage the integration of ESs with market-driven incentives. For example, previous studies have demonstrated the feasibility of incorporating bamboo forests into carbon credit trading frameworks (Nath et al. 2015; Wu et al. 2015), as well as the potential of payment for ecosystem services (PES) schemes to yield dual benefits in conservation and social welfare (Chen et al. 2020). On the other hand, the analysis also highlights a pressing need for greater attention and structural support for research on bamboo ESs to address the current fragmentation in authorship and the lack of long-term research initiatives. We recommend that national agencies or research centers establish dedicated units focused on bamboo. These units should be tasked with funding the development of a cohesive academic community, which is essential for informed science-based policy development. Notable examples of such institutional efforts include the Center for International Forestry Research (CIFOR) and the International Network for Bamboo and Rattan (INBAR), both of which have significantly advanced research and fostered international collaboration on bamboo resources.

The second key finding highlights that most studies are concentrated in regions that closely align with the global distribution of bamboo forests. This suggests that research efforts have been effectively directed toward areas rich in bamboo ecosystem resources, thereby supporting the development of more locally tailored bamboo forest management policies. In particular, the prominent role of Chinese researchers in this field is notable. China has witnessed a rapid expansion in ES-related research over recent decades (Jiang 2017), alongside a rising number of bamboo-focused studies conducted by Chinese researchers (Zhang et al. 2024). Meanwhile, a substantial amount of research conducted in developing countries is led by Western researchers, who may face significant challenges due to limited familiarity with the local context. This can increase the risk of sampling bias, particularly for researchers from outside the country who may lack in-depth knowledge of local social structures, cultural practices, or community dynamics. Without sufficient engagement with local stakeholders, there is a heightened risk that research findings may misrepresent or overlook key local perspectives. Moreover, issues related to research ethics and power dynamics must be carefully addressed, especially in contexts where external researchers hold greater institutional or financial authority. Ensuring that the voices and perspectives of local communities are meaningfully included requires not only participatory methods but also a critical reflection on how research agendas are set, who benefits from the findings, and how knowledge is co-produced.

Following the research into keyword analysis, three main thematic focuses emerge from the co-occurrence clusters and their associated literature. These focuses provide deeper insight into how bamboo ESs are studied and managed across different regions and methodological traditions. The first focus, enhancing understanding through quantitative ESs, emphasizes the importance of measurable indicators such as carbon sequestration and biodiversity conservation (Yuen et al. 2017). This scientific approach can support the development of evidence-based management strategies that optimize bamboo’s benefits. The second focus incorporates human dimensions, highlighting qualitative factors such as attitudes and adaptive behaviors. This perspective underscores the importance of local communities’ perceptions and adaptive practices for bamboo. These factors contribute to the development of ecologically sustainable and socially equitable management strategies, particularly in regions where livelihoods are closely linked to bamboo resources (Lee 2021). The third focus involves informed decision-making by economic assessment. Through the valuation of bamboo ESs, this approach enables the integration of ESs into national and regional economic planning frameworks (Zandebasiri et al. 2023). It also strengthens the policy relevance of bamboo conservation by making its benefits more visible and quantifiable and may attract targeted investments to promote long-term sustainability.

Fourth, our study identifies the pivotal role played by Chinese researchers in advancing the quantification of bamboo ESs, standing in contrast to the qualitative methodologies used by scholars from Western countries. This divergence has led to the emergence of two distinct research paradigms. However, each methodology has its own (dis)advantages. Quantitative studies enable researchers to identify broad patterns and trends, which are crucial for informing management policies at national and subnational levels. In contrast, qualitative research methods offer a deep understanding at the local scale, which is critical to aligning ecological goals with social priorities. Such approaches help develop management strategies based on local experiences and consider the complex socioeconomic factors influencing bamboo management (Busch et al. 2012). We therefore suggest the development of interdisciplinary research teams that integrate expertise across the natural and social sciences. By considering both biophysical and socioeconomic dimensions, these teams can create a holistic evaluation model combining quantitative and qualitative indicators to assess the full spectrum of bamboo ES values. This integrated approach will enhance the comprehensiveness of bamboo management strategies.

Fifth, the limited attention given to bamboo’s cultural ESs in existing literature underscores inherent challenges in examining these services (Hernández-Morcillo et al. 2013). This research gap may be attributed to the inherent difficulty in collecting data on cultural ES and the methodological challenges associated with applying quantitative approaches. Consequently, qualitative research is essential to complement existing quantitative studies and provide deeper insights into cultural ES. Cultural ESs may encompass educational and recreational activities related to bamboo forests, as well as emotional connections and traditional values associated with bamboo within local cultures. Accurately capturing and comprehending these cultural dimensions typically requires ethnography and community-based interviews. Recognizing and incorporating the cultural significance of bamboo into management strategies are crucial steps to ensure these values are not overlooked. Adopting such an approach contributes to more comprehensive assessments of bamboo’s overall ESs, supporting more effective and inclusive bamboo forest management practices.

Finally, the findings regarding synergies and trade-offs in bamboo ESs are crucial for formulating effective bamboo management strategies. These insights guide managers to balance bamboo’s ecological conservation and economic utilization, particularly considering long-term impacts and future projections. For instance, the degradation of bamboo might affect biodiversity or carbon storage (Yuan et al. 2023), and bamboo invasion might impact litter and soil properties (Luo et al. 2023; Sardar et al. 2023; Wei et al. 2021; Chen et al. 2021), necessitating a balanced approach between these ecological benefits and economic gains. Additionally, the interaction of bamboo ESs exhibits spatial heterogeneity across different regions, requiring management strategies to be tailored to specific geographical and environmental conditions. However, our systematic review found that only a limited number of studies explicitly examined trade-offs or synergies associated with bamboo. Due to this scarcity of evidence, we could not conduct a thorough analysis across different spatial scales (e.g., farm, landscape, and regional levels). Integrating these complex interactions is vital for developing more effective and sustainable bamboo management strategies, underscoring the importance of balancing the utilization and protection of bamboo resources.

### Limitations

This study has several limitations that should be considered when interpreting its findings. First, the exclusive reliance on the Scopus database for literature identification may have constrained the comprehensiveness of the review. Although Scopus is a widely used and reputable source, depending solely on a single database could limit the diversity and depth of the literature included. For example, working papers from organizations like CIFOR and INBAR can provide valuable insights that are often excluded from conventional database searches. Using these additional sources may lead to a more thorough and representative review of the existing literature. Additionally, the review was limited to studies published in specific languages. This language restriction may have led to the exclusion of relevant research published in other languages, particularly in regions such as Southeast Asia, where important studies may only be available in local languages.

Furthermore, this systematic review was conducted using a predefined “ecosystem service” set of keywords. However, some relevant studies may not explicitly use this term despite addressing ES-related themes. As a result, potentially valuable contributions might have been missed. Employing other search queries may help increase the number of relevant records identified. For instance, several emerging terms closely related to ESs have appeared recently, such as “nature’s contributions to people” (NCP), which could help capture a broader range of literature.

Lastly, the process of selecting and evaluating literature inevitably involves a degree of subjectivity. This subjectivity arises from decisions made during literature screening, keyword selection, and data extraction and extends to evaluating literature quality, relevance, and significance. To mitigate potential bias introduced by individual judgment, we recommend involving more independent reviewers in future review processes. Additionally, to address the variation in study quality, future research could consider employing a weighted review method to improve the robustness of synthesis outcomes.

Several important studies from Asian countries published in recent years, such as those by Ayer et al. (2023) and Paudyal et al. (2022), were not captured by the current review strategy. This is because incorporating these studies post hoc would compromise the methodological transparency and reproducibility expected in a systematic review. Future research can consider conducting semi-systematic or narrative review approaches to supplement keyword-based strategies. These methods enable more targeted exploration of key journals, particularly those focused on domain-specific themes. One example is *Advances in Bamboo Science*, which consistently publishes research on bamboo and its related ESs.

## Conclusion

This systematic literature review comprehensively synthesizes current knowledge and research trends on the ESs associated with bamboo. The significance of this review lies in its systematic consolidation of fragmented knowledge on bamboo ESs, which informs both forest management and academic research. Mapping existing evidence and identifying critical gaps offers researchers a foundation to build upon prior insights, generate new knowledge to support bamboo management and design future studies on bamboo’s ESs.

Based on the analysis of 56 selected publications, researchers have shown increased interest in bamboo ESs compared to previous periods. Policymakers and managers are encouraged to utilize these latest research findings to enhance sustainable management strategies, such as integrating bamboo forests into carbon trading and PES programs. To facilitate continuity and collaboration in bamboo ES research, we recommend establishing dedicated units to address the fragmented nature of authorship, institutional affiliations, and funding sources.

On the other hand, although research is predominantly concentrated in regions with abundant bamboo resources, Chinese researchers notably contributed 22 of the reviewed publications, significantly surpassing other individual countries. Nearly all studies conducted by Chinese researchers focus on China, whereas researchers from developed countries generally conduct studies in developing countries outside of China. Keyword analysis identified three primary research domains: quantitative assessment, qualitative investigation, and economic valuation. For quantitative studies, plot sampling emerged as the most commonly used method for data collection. Chinese researchers, benefiting from the greater availability of public datasets, frequently employed quantitative analysis and modeling techniques, such as the InVEST model. Conversely, researchers from other countries often adopted qualitative methods due to data scarcity in developing regions. Integrating interdisciplinary teams that combine quantitative and qualitative methodologies is recommended to achieve a more comprehensive evaluation of the ecological and socioeconomic values of bamboo forests.

Among the ES categories analyzed, regulation and maintenance services received the most attention. In contrast, cultural services were comparatively underrepresented, highlighting a significant research gap. This lack of attention underscores the necessity for further exploration into cultural services to ensure these dimensions are adequately integrated into management strategies and to prevent the undervaluation of overall ESs. Additionally, most studies identified ESs individually rather than exploring their interactions. Although some research illustrated relationships between two ESs through matrices, few publications addressed spatial heterogeneity and temporal dimensions. We suggest that clarifying the complex interactions among different ESs is crucial for effectively balancing synergies and trade-offs among these services.

To sum up, this review provides critical insights for improving bamboo forest management and identifies key research directions that warrant further in-depth investigation.

## Data Availability

The data that support the findings of this study are available from the corresponding author upon reasonable request.

## Appendix

**Table 2:** Simple descriptors for each CICES code

Section	CICES code	Simple descriptor
Provisioning	1.1.1.1	Any crops and fruits grown by humans for food; food crops
	1.1.1.2	Material from plants, fungi, algae or bacteria that we can use
	1.1.1.3	Plant materials used as a source of energy
	1.1.2.1	Plants that are cultivated in fresh or salt water that we eat
	1.1.2.2	Plants that are cultivated in fresh or salt water that we can use as a material
	1.1.2.3	Plants that are cultivated in fresh or salt water that we can use as an energy source
	1.1.5.1	Food from wild plants
	1.1.5.2	Materials from wild plants
	1.1.5.3	Materials from wild plants, fungi and algae used for energy
	1.2.1.1	Seed collection
	1.2.1.2	Plants. fungi or algae that we can use for breeding
	1.2.1.3	Genetic material from wild plants. fungi or algae that we can use
	1.3.X.X	Other types of provisioning service from biotic sources
Regulation & Maintenance	2.1.1.1	Decomposing wastes
	2.1.1.2	Filtering wastes
	2.1.2.1	Reducing smells
	2.1.2.2	Reducing noise
	2.1.2.3	Screening unsightly things
	2.2.1.1	Controlling or preventing soil loss
	2.2.1.2	Stopping landslides and avalanches from harming people
	2.2.1.3	Regulating the flows of water in our environment
	2.2.1.4	Protecting people from winds
	2.2.1.5	Protecting people from fire
	2.2.2.1	Pollinating our fruit trees and other plants
	2.2.2.2	Spreading the seeds of wild plants
	2.2.2.3	Providing habitats for wild plants and animals that can be useful to us
	2.2.3.1	Controlling pests and invasive species
	2.2.3.2	Controlling disease
	2.2.4.1	Ensuring soils form and develop
	2.2.4.2	Ensuring the organic matter in our soils is maintained
	2.2.5.1	Controlling the chemical quality of freshwater
	2.2.5.2	Controlling the chemical quality of salt water
	2.2.6.1	Regulating our global climate
	2.2.6.2	Regulating the physical quality of air for people
	2.3.X.X	Other types of regulation and maintenance service by living processes
Cultural	3.1.1.1	Using the environment for sport and recreation; using nature to help stay fit
	3.1.1.2	Watching plants and animals where they live; using nature to destress
	3.1.2.1	Researching nature
	3.1.2.2	Studying nature
	3.1.2.3	The things in nature that help people identify with the history or culture of where they live or come from
	3.1.2.4	The beauty of nature
	3.2.1.1	Using nature to as a national or local emblem
	3.2.1.2	The things in nature that have spiritual importance for people
	3.2.1.3	The things in nature used to make films or to write books
	3.2.2.1	The things in nature that we think should be conserved
	3.2.2.2	The things in nature that we want future generations to enjoy or use
	3.3.X.X	Other characteristics of living systems that have cultural significance

Source: Haines-Young and Potschin (2018)

**Table 3:** The indicators of ES recognized by the 56 selected publications

CICES code	Indicators	Reference
1.1.1.2	Culms/timber	Embaye (2001)^**^; Okubo et al. (2010)^*^; Gao et al. (2013)^*^; Zhang et al. (2016)^**^; Dai et al. (2017)^**^; Pande et al. (2017)^**^; Houdanon et al. (2018)^**^; Ahammad et al. (2019)^*^; Maosew et al. (2019)^*^; Yeasmin et al. (2021)^*^; Lee (2021)^**^; Dai et al. (2022)^**^
	Non-timber forest products	Gao et al. (2013)^*^; Zhang et al. (2016)^**^; Gurung et al. (2021)^**^; Ma et al. (2022)^**^
1.1.5.1	Food/bamboo shoot	Embaye (2001)^**^; Zhang et al. (2016)^**^; Ahammad et al. (2019)^*^; Gurung et al. (2021)^**^; Lee (2021)^**^; Yeasmin et al. (2021)^*^; Ndavaro et al. (2023)^**^
1.1.5.2	Construction and furniture applications/thatching grass	Embaye (2001)^**^; Okubo et al. (2010)^*^; Ingale and Deore (2015)^**^; Zhang et al. (2016)^**^; Houdanon et al. (2018)^**^; Ahammad et al. (2019)^*^; Ndavaro et al. (2023)^**^
1.1.5.3	Energy/fuel	Embaye (2001)^**^; Okubo et al. (2010)^*^; Ahammad et al. (2019)^*^; Maosew et al. (2019)^*^; Lee (2021)^**^; Gurung et al. (2021)^**^; Ndavaro et al. (2023)^**^
1.3.X.X	Support for agriculture	Okubo et al. (2010)^*^; Zhang et al. (2016)^**^; Houdanon et al. (2018)^**^; Ndavaro et al. (2023)^**^
	Handicraft/bamboo product/broom grass	Zhang et al. (2016)^**^; Houdanon et al. (2018)^**^; Ahammad et al. (2019)^*^; Gurung et al. (2021)^**^; Ndavaro et al. (2023)^**^
	Fodder/forage	Embaye (2001)^**^; Zhang et al. (2016)^**^; Ahammad et al. (2019)^*^; Gurung et al. (2021)^**^
	Medicine	Embaye (2001)^**^; Houdanon et al. (2018)^**^; Lee (2021)^**^; Ndavaro et al. (2023)^**^;
	Biomass	Shinohara et al. (2014)^***^; Sujarwo (2016)^**^; Yu et al. (2016)^***^; Houdanon et al. (2018)^**^; Nath et al. (2018)^**^; Maosew et al. (2019)^*^; Yeasmin et al. (2021)^*^; Padgurschi et al. (2021)^**^; Abebe et al. (2021)^*^; Yebeyen et al. (2022)^**^; Ma et al. (2022)^**^
	Freshwater	Lee (2021)^**^
2.1.1.2	Environmental cleaning	Embaye (2001)^**^
2.1.2.1	Anion/air purifying/fresh air regulation	Wang et al. (2011)^**^; Hu et al. (2013)^***^; Liu et al. (2013)^**^; Gao et al. (2013)^*^; Huang et al. (2018)^**^; Ahammad et al. (2019)^*^; Cui et al. (2019)^*^; Lee (2021)^**^
2.2.1.1	Soil conservation/protection/retention/erosion control/fix soil	Embaye (2001)^**^; Wang et al. (2011)^**^; Hu et al. (2013)^***^; Gao et al. (2013)^*^; Liu et al. (2013)^**^; Shinohara et al. (2014)^***^; Sujarwo (2016)^**^; Dai et al. (2017)^**^; Pande et al. (2017)^**^; Huang et al. (2018)^**^; Ahammad et al. (2019)^*^; Cui et al. (2019)^*^; Maosew et al. (2019)^*^; Lee (2021)^**^; Dai et al. (2022)^**^; Feng et al. (2022)^*^; Ma et al. (2022)^**^
2.2.1.2	Probability of surface collapse	Shinohara et al. (2014)^***^
2.2.1.3	Soil water storage	Pan et al. (2012)^**^; Maosew et al. (2019)^*^; Muñoz-López et al. (2021)^**^; Zhuang et al. (2022)^***^
	Water conservation/storage/water-holding capacity	Embaye (2001)^**^; Saxena et al. (2002)^*^; Wang et al. (2011)^**^; Hu et al. (2013)^***^; Gao et al. (2013)^*^; Shinohara et al. (2014)^***^; Sujarwo (2016)^**^; Huang et al. (2018)^**^; Maosew et al. (2019)^*^; Cui et al. (2019)^*^
	Water yield	Dai et al. (2017)^**^; Dai et al. (2022)^**^; Feng et al. (2022)^*^
	Water regulation	Liu et al. (2013)^**^; Cui et al. (2019)^*^
	Reduce flood	Lin et al. (2017)^**^; Pan et al. (2012)^**^; Shinohara et al. (2014)^***^; Ma et al. (2022)^**^
2.2.1.4	Against wind	Embaye (2001)^**^
2.2.2.1	Crop pollination	Ahammad et al. (2019)^*^
2.2.2.3	Biodiversity conservation	Wang et al. (2011)^**^; Hu et al. (2013)^***^; Gao et al. (2013)^*^; Liu et al. (2013)^**^; Huang et al. (2018)^**^; Cui et al. (2019)^*^; Yeasmin et al. (2021)^*^; Lee (2021)^**^
	Panda habitat	Li et al. (2017)^**^
	Red panda habitat	Panthi et al. (2017)^**^; Bhatta et al. (2022)^**^; Karki et al. (2021)^**^
	Habitat for species	Lee (2021)^**^; Ma et al. (2022)^**^
2.2.3.1	Biological control/pest and disease control	Houdanon et al. (2018)^**^; Ahammad et al. (2019)^*^
2.2.4.1	Soil formation	Embaye (2001)^**^
2.2.4.2	Soil nutrition content	Maosew et al. (2019)^*^
	Nitrogen accumulation	Saxena et al. (2002)^*^; Wang et al. (2011)^**^; Liu et al. (2013)^**^; Cui et al. (2019)^*^; Zhuang et al. (2022)^***^; Huang et al. 2018^**^; Ma et al. (2022)^**^
	Phosphorus accumulation	Wang et al. (2011)^**^; Liu et al. (2013)^**^; Huang et al. (2018)^**^; Cui et al. (2019)^*^; Zhuang et al. (2022)^***^; Ma et al. (2022)^**^
	Decreased loss of potassium	Wang et al. (2011)^**^; Liu et al. (2013)^**^; Huang et al. (2018)^**^; Cui et al. (2019)^*^
	Nutrients accumulation	Wang et al. (2011)^**^; Hu et al. (2013)^***^; Huang et al. (2018)^**^; Cui et al. (2019)^*^
	Nutrient cycle	Gao et al. (2013)^*^
	Nitrogen/phosphorus/potassium/fertility/organic carbon maintenance	Saxena et al. (2002)^*^; Wang et al. (2011)^**^; Liu et al. (2013)^**^; Yu et al. (2016)^***^; Huang et al. (2018)^**^; Ahammad et al. (2019)^*^; Maosew et al. (2019)^*^; Cui et al. (2019)^*^; Xiao et al. (2023)^***00^
	Soil pH	Xiao et al. (2023)^***^
2.2.5.1	Water purification	Liu et al. (2013)^**^; Ahammad et al. (2019)^*^; Lee (2021)^**^
2.2.6.1	Carbon sequestration/storage/fixation/accumulation	Saxena et al. (2002)^*^; Hu et al. (2013)^***^; Gao et al. (2013)^*^; Liu et al. (2013)^**^; Li et al. (2015)^***^; Sujarwo (2016)^**^; Yu et al. (2016)^***^; Dai et al. (2017)^**^; Houdanon et al. (2018)^**^; Nath et al. (2018)^**^; Huang et al. (2018)^**^; Cui et al. (2019)^*^; Wu et al. (2020)^***^; Li et al. (2020)^**^; Yeasmin et al. (2021)^*^; Padgurschi et al. (2021)^**^; Abebe et al. (2021)^*^; Lee (2021)^**^; Muñoz-López et al. (2021)^**^; Zhuang et al. (2022)^***^; Dai et al. (2022)^**^; Yu et al. 2016^***^; Yebeyen et al. (2022)^**^; Feng et al. (2022)^*^; Ma et al. (2022)^**^
	Oxygen release	Wang et al. (2011)^**^; Hu et al. (2013)^***^; Gao et al. (2013)^*^; Liu et al. (2013)^**^; Huang et al. (2018)^**^; Cui et al. (2019)^*^
	Climate regulation	Gao et al. (2013)^*^; Yeasmin et al. (2021)^*^
	Cooling effect	Yeasmin et al. (2021)^*^
2.2.6.2	Dust-blocking/absorb PM_10_/absorb PM_2.5_/TSP	Wang et al. (2011)^**^; Gao et al. (2013)^*^; Huang et al. (2018)^**^; Cui et al. (2019)^*^;
	Absorption of SO_2_	Wang et al. (2011)^**^; Liu et al. (2013)^**^; Huang et al. (2018)^**^; Cui et al. (2019)^*^
	Absorption of HF	Wang et al. (2011)^**^; Cui et al. (2019)^*^; Huang et al. (2018)^**^
	Absorption of NO_*x*_	Wang et al. (2011)^**^; Huang et al. (2018)^**^; Cui et al. (2019)^*^
2.3.X.X	Land rehabilitation	Sujarwo (2016)^**^
	Prevent disaster/hazard	Gao et al. (2013)^*^; Yeasmin et al. (2021)^*^; Lee (2021)^**^
	Against sun	Embaye (2001)^**^
	Coastal protection	Baul et al. (2021)^**^
3.1.1.1	Recreation	Embaye (2001)^**^
3.1.2.2	Biophilia	Yeasmin et al. (2021)^*^
3.1.2.3	Folklore	Gao et al. (2013)^*^
	Culture	Gao et al. (2013)^*^; Zhang et al. (2016)^**^; Houdanon et al. (2018)^**^
	Ritual products	Gurung et al. (2021)^**^
3.1.2.4	Aesthetic benefit/value	Embaye (2001)^**^; Gao et al. (2013)^*^; Ahammad et al. (2019)^*^; Yeasmin et al. (2021)^*^
	Landscape beauty	Lee (2021)^**^
3.2.1.2	Spiritual benefit	Ahammad et al. (2019)^*^
	Spirits and religious	Gao et al. (2013)^*^; Lee (2021)^**^
3.2.1.3	Relax and stress reduction	Yeasmin et al. (2021)^*^
	Forest recreation	Liu et al. (2013)^*^; Cui et al. (2019)^*^
	Biophilia	Yeasmin et al. (2021)^*^
	Worship	Ndavaro et al. (2023)^**^
3.2.2.2	Eco-tourism	Gao et al. (2013)^*^; Zhang et al. (2016)^**^; Sujarwo (2016)^**^

Note: ^*^ES from general forest (including bamboo); ^**^unknown, other or all type(s) of bamboo; ^***^Moso bamboo. The types related to animals (CICES code was 1.1.3.1–⁠1.1.4.3, 1.1.6.1–⁠1.1.6.3, and 1.2.2.1–⁠1.2.2.3) and abiotic (CICES code was 4.2.1.1–⁠6.3.X.X) were omitted. For the meaning of each CICES code, see Appendix Table 2

## Declarations

During the preparation of this work, the authors used ChatGPT and Grammarly to improve language readably. After using these tools/services, the authors reviewed and edited the content as needed, and take full responsibility for the content of the publication.
